# Nutritional Value and Structure Characterization of Protein Components of *Corylus mandshurica Maxim*

**DOI:** 10.3390/molecules28176355

**Published:** 2023-08-30

**Authors:** Yanli Hou, Jie Ding, Qingqi Guo, Na Zhang

**Affiliations:** 1 College of Life Science, Northeast Forestry University, Harbin 150040, China; houyanli171@163.com (Y.H.); dj1535442944@163.com (J.D.); 2 College of Food Engineering, Harbin University of Commerce, Harbin 150028, China

**Keywords:** *Corylus mandshurica Maxim*, Osborne method, essential amino acids, nutritional value, structure characterization

## Abstract

Alternative protein sources for the human diet may help overcome the growing food pressure. Plants with abundant resources and high protein content are potential sources. In this article, graded proteins and isolated proteins from *Corylus mandshurica Maxim* kernels were extracted by the Osborne procedure and the alkali-solution and acid-isolation method, respectively, and the contents of the five proteins, and the differences in nutritional value and structural properties of the main proteins, were investigated. Amino acid analysis revealed that the total essential amino acids in the five proteins ranged from 249.58 to 324.52 mg/g. The essential amino acid profiles in the proteins were similar to those of FAO/WHO except for the alcohol-soluble protein. The essential amino acid indices ranged from 58.59 to 72.19 and the biological values ranged from 52.16 to 66.99, and the highest nutritional indices were found for the isolate and water-soluble protein, which were 41.68 and 55.78, respectively. The molecular weight pattern distribution of the protein isolates of the *Corylus mandshurica Maxim* kernel was more similar to that of the water-soluble proteins by SDS–PAGE. The β-sheet and α-helix were the main secondary structures in the two protein fractions. The fluorescence spectra showed that the maximum fluorescence intensity of the two proteins and their λmax were also somewhat different. From the perspective of microscopic morphology, the two proteins are mainly compact and irregular lamellar structures, but the surface of the water-soluble protein is more flat and regular. Both proteins have good solubility, and the water-soluble protein has higher solubility. In general, the protein isolates of the *Corylus mandshurica Maxim* kernel and the water-soluble protein showed their potential as plant protein resources.

## 1. Introduction

In recent years, the large-scale planting of main food crops has put enormous pressure on the ecological environment. To achieve the sustainable development of agriculture, ecological protection, and the balanced development of agricultural modernization, the concept of a wide range of food that requires heat and protein from cultivated land, grassland, forests and oceans, plants, animals and microorganisms, and the all-around and multi-channel development of food resources should be actively practiced and developed. Furthermore, in the face of accelerated urbanization and increased population aging, the role of proteins in a healthy diet and the contribution of aging is gaining attention [1,2]. Compared to animal proteins, plant proteins are cheaper to produce, require less land, and have less impact on climate change with smaller GHG emissions, and the development of plant proteins can fully utilize agricultural by-products and waste resources [3,4]. At the same time, plant-based proteins have a lower probability of infection and contamination, are less restrictive concerning cultural and religious dietary practices, and are more acceptable to vegan consumers [3,5,6]. Today, several types of plants such as legumes, cereals, pseudo-cereals, seeds, nuts, etc., have been widely used as alternative plant proteins in research [7]. Considered an excellent choice for human nutrition because of their high protein and amino acid content, legumes are widely processed and studied and have become a major source of plant protein in developing countries. Represented by soybeans and chickpeas, these resources are now being developed and used in products such as bakery products, vegetable protein drinks, meat analogs, etc., with the advantage of being relatively low-cost and sustainable [8,9,10]. Rice, sorghum, and wheat are typical cereal protein sources, and pseudo-cereal protein sources are mainly amaranth, quinoa, and buckwheat [11,12]. These resources have a high content of essential amino acids and are particularly rich in sulfur-containing amino acids, which are highly bioavailable and more easily digested and utilized by the human body [13,14]. As a major food crop, this type of vegetable protein is mainly consumed in the form of flour or grains/seeds and is also used in salads, cakes, bread, etc. [15,16]. It not only provides people with basic dietary energy but also increases fatty acid, fiber, and mineral intake, with greater prospects for application [12,16]. Common sources of seed protein are mainly flaxseeds and chia seeds, which are high-quality proteins. However, the lysine content is low, so it must be consumed in conjunction with other proteins to overcome this amino acid deficiency [17,18]. Nuts are another source of vegetable protein, as well as being high in fatty acids and fiber, and contain many minerals and natural active substances, which play an important role in regulating blood lipids, preventing and controlling coronary heart disease, and enhancing brain memory. Currently, most nuts are consumed as casual snacks or included in food products, and some are used in dairy products [19,20]. In addition to the more prevalent plant protein resources mentioned above, another group of plant proteins has also attracted the attention of researchers from another perspective, i.e., the wild protein plant resources. This kind of protein resource often belongs to the local specialties, but with increased economic development, the increase in consumption level, and the diversification of food, this kind of resource is often neglected due to the change in people’s dietary habits. This type of resource is a rich source of carbohydrates, proteins, amino acids, and other nutrients, and the resource is well adapted to the environment and is free of pesticides and chemical pollution, thus contributing to the realization of ecologically sustainable development [14,21]. Its development and utilization will also increase the variety of alternative plant protein resources, meet the dietary and nutritional needs of local people, and improve human health and living standards.

*Corylus mandshurica Maxim* is a shrubby plant of the genus Hazel in the family Birchaceae. It has a sweet taste, calm nature, and non-toxicity, which helps the health of the spleen and stomach, and brightens the eyes. Mainly distributed in northeastern China, North China, Northwest China, and other regions, it is produced in Heilongjiang Province, Xiaoxing’an Mountains, Zhang Guangcai Mountains, Laoyao Mountains, and Wandashan mountainous areas, and in the wild. It has a strong ability to resist cold, can grow on poor land, and is adaptable; its fruit skin is thin and its kernel quality is good, rich in a variety of amino acids, and has a high nutritional value [22,23]. There are fewer specific studies on the protein content and nutritional value of *Corylus mandshurica Maxim*, but studies have shown that hazelnut protein content is about 16–18% [24]. Wei et al. [25] found that the amino acids in the four graded proteins and isolate proteins were closer to the FAO/WHO recommended patterns after nutritional analysis of the protein fractions of flat European hazelnut. This indicates that hazelnut is a higher protein plant and its nutritional value is more in line with human dietary patterns. Therefore, in this experiment, the *Corylus mandshurica Maxim* from the Xiaoxing’an Mountains of northeastern China was used as raw material to prepare four kinds of graded protein and isolated protein by the Osborne procedure and the alkali-solution and acid-isolation method, and the content of the five kinds of protein and their amino acid composition was determined. The nutritional value of different fractions of *Corylus mandshurica Maxim* kernel proteins was analyzed by FAO/WHO and the whole egg recommendation model, and the structure of the main proteins was determined, in order to provide more sources of alternative plant proteins and to provide a theoretical basis for the development and utilization of *Corylus mandshurica Maxim* resources.

## 2. Results

### 2.1. Chemical Composition of Corylus mandshurica Maxim Kernel and Defatted Corylus mandshurica Maxim Kernel Flours

Table 1 shows that the fat content of the *Corylus mandshurica Maxim* kernel raw material is high, so the ash and protein content will be significantly increased after degreasing treatment. The crude protein content of the raw material from *Corylus mandshurica Maxim* kernel is 21.89%, which is similar to the protein content in most nuts, but higher than the protein content in cereals and lower than the protein content in beans [17,26]. After defatting, the protein content of the DCF is about 50%, which can be used as a good protein raw material. It can also be seen from the table that the content of the four basic components in the *Corylus mandshurica Maxim* kernel and the DCF did not achieve mass conservation, which may be because the *Corylus mandshurica Maxim* kernel also contains a certain amount of starch, soluble sugar, and cellulose [27].

### 2.2. Extraction Rate and Protein Content of Protein Isolates of Corylus mandshurica Maxim Kernel and Graded Proteins from DCF

The extraction rates of the five proteins in the DCF varied widely. Where the extraction rate of the CPI was 49.01%, the extraction rate of the WS was the highest among the graded proteins at 63.43%, and the SS (9.96%) and the LS (3.50%) decreased sequentially, while the AS protein had the lowest extraction rate at only 1.34%. This ratio is the same as that of Flat-European hazelnut hybrid hazelnut protein [25]. There may be some differences in the protein content due to different extraction methods. The content of the proteins was determined after freeze-drying and we found that the content of the AS was low at 38.46%; the content of the WS protein, the content of the SS protein, and the LS content were similar at 67.45%, 72.35%, and 74.49%, respectively. The content of the CPI was higher at 92.53%, probably due to the removal of some impurities during the acid precipitation process.

### 2.3. Amino Acid Composition of the Five Proteins in Corylus mandshurica Maxim Kernel

The nutritional value of the proteins can be assessed by determining the composition and content of amino acids. The amino acid content of the CPI and of the four graded proteins extracted from the DCF was analyzed and determined using the automatic amino acid autoanalyzer, and the results are shown in Table 2. As a reference, the Food and Agriculture Organization/World Health Organization (FAO/WHO) recommended modes of the essential amino acids for adults are also given in Table 2. Tryptophan is not listed in the table as it was acid-hydrolyzed and destroyed [28].

Table 2 shows that all four of the five proteins contain 17 amino acids, except for the AS, which does not contain arginine. There is a certain difference in the total amount of amino acids in each protein, mainly because the content of each amino acid in the AS is quite different from other proteins. However, the content of methionine and cystine in gliadin is higher than that of other proteins, accounting for 47.03% of the total amino acids, which is similar to the AS in corn [29]. The content of amino acids in the LS is similar. The content of the other three proteins is high, and the main amino acids are glutamic acid, arginine, aspartic acid, and leucine. The total amount of the four amino acids accounts for 53.63%, 48.92%, and 52.85% of the total amino acid content of each protein.

EAA are the amino acids that the body must obtain from the diet. The total amount of EAA in the five types of protein ranges from 249.58 to 324.52 mg/g, with a relatively small difference. AS and LS were higher than the FAO/WHO standards of 40% and 0.6 in the results regarding EAA/TAA and EAA/NEAA [30]; the ratio of EAA/NEAA is relatively prominent, at 4.34 and 1.08, respectively, which is closely related to the relatively high proportion of methionine. The amino acid ratios of the other proteins are less than that specified by FAO/WHO standards, but they are all around 30% and 0.4, which is closer to Juglandaceae. [31]. In addition, a comparison of essential amino acids with some food crops showed that, except for some differences in the AS content, the essential amino acid content of the other proteins is close to or higher than that found in peanut, soybean, and wheat proteins [32], so *Corylus mandshurica Maxim* can be considered an important source of natural plant protein and is expected to be an ideal replacement protein for food crops.

### 2.4. Assessment of the Nutritional Value of Five Proteins in Corylus mandshurica Maxim Kernel Proteins

The nutritional value of food depends mainly on the type, quantity, and composition of essential amino acids, and the theory of amino acid balance suggests that the closer the ratio of amino acid composition is to that of the FAO/WHO model, the better the amount of protein [33]. In addition to the AS, the contents of Phe + Tyr, valine, leucine, and isoleucine in the seven essential amino acids of each protein are close to the FAO/WHO standard, indicating that these four amino acids in *Corylus mandshurica Maxim* kernel proteins play an important role in the nutritional value of protein. Threonine and lysine were low and deviated from the standard pattern among the amino acids of each protein. As shown in Table 3, the AAS and CS calculations show that similar to cereal proteins such as wheat, barley and rice, these two amino acids are also the first and second limiting amino acids in the five proteins [34]. The content of Met + Cys in all proteins is higher than the standard model, and it is the highest content of amino acid in other proteins except the CPI. Therefore, the *Corylus mandshurica Maxim* kernel proteins can be regarded as a food with rich sulfur content.

The EAAI takes into account the ratio of all EAA in the protein to all EAA in the model protein; biological value is considered to be the degree of protein digestion and utilization [35]. Taking the whole egg as a reference [36], the closer the value of EAAI is to 100, the closer the ratio of EAA in the protein is to the egg, and the higher the nutritional value will be [37]. It can be seen from Table 4 that the EAAI and BV of the five proteins are ordered as WS, CPI, SS, LS, and AS from high to low. Although there is a certain difference in the proportion in eggs, the EAAI and biological value values of the other proteins are similar to those of corn cultivars, except that the AS is low [38]. To comprehensively consider the content and amino acid composition of the *Corylus mandshurica Maxim* kernel proteins, the NI of each protein was calculated. The NI values were found to be low not only for the AS but also for the SS and the LS. It shows that even though the content of each amino acid in the protein is similar to the standard model, if the content of this protein is low, it still cannot give full play to its nutritional value.

Overall, among the five proteins, the nutritional value of the AS deviated far from the standard model, and among the remaining four proteins, the WS and the SS were closer to the standard nutritional model, and these two proteins had a higher content in *Corylus mandshurica Maxim*, which had a greater potential for development, and therefore were selected to continue the study of the structural properties of these two proteins.

### 2.5. Structural Properties

#### 2.5.1. SDS–PAGE Analysis

**Figure 1:**
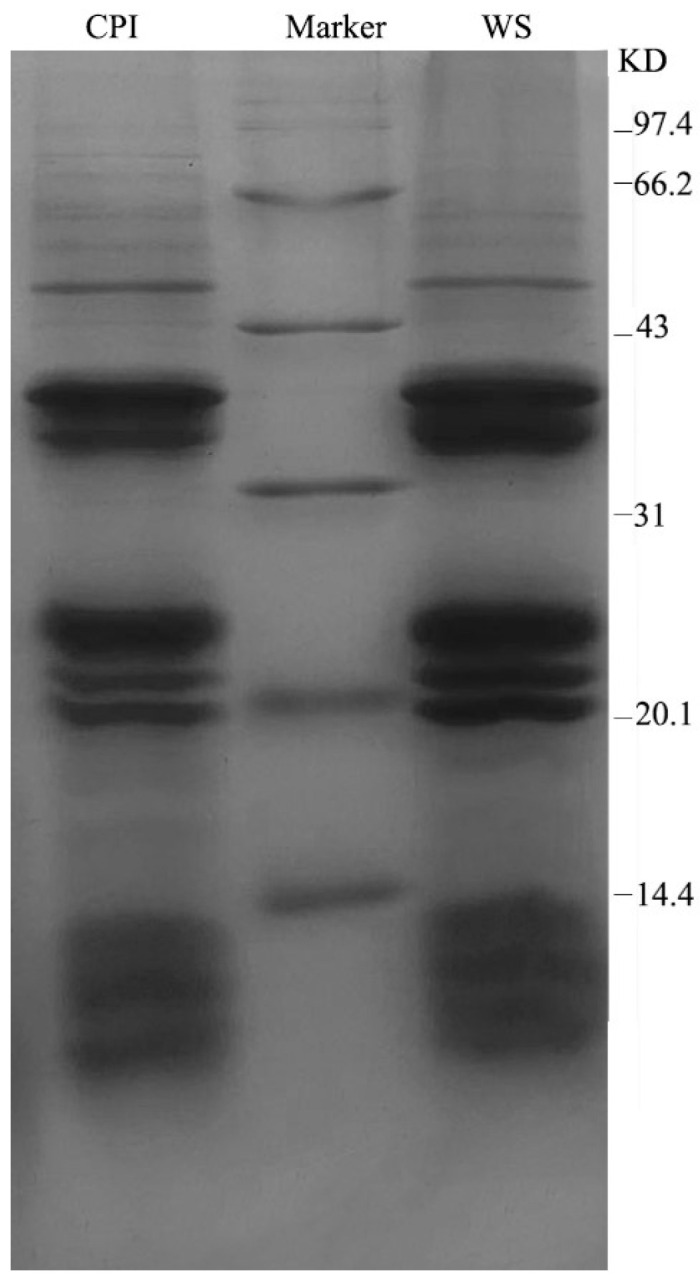
Electrophoresis of *Corylus mandshurica Maxim* kernel proteins.

As can be seen from Figure 1, the molecular weights of the CPI and the WS are relatively small, both distributed within 97.4 KD. The molecular weight of the CPI is mainly distributed at about 48 KD, 38–39 KD, 35–36 KD, 24–25 KD, 22 KD, 19 KD, and below the range of 14.4 KD. Except for the 48 KD band, the other bands are wider and darker, indicating that these subunits are more abundant and account for the main components. Except for some differences in the content of subunit bands, the WS and the CPI showed similar patterns, suggesting that there may also be many similarities in the composition and properties of the two proteins.

#### 2.5.2. Fourier Transform Infrared Spectroscopic Analysis

**Figure 2:**
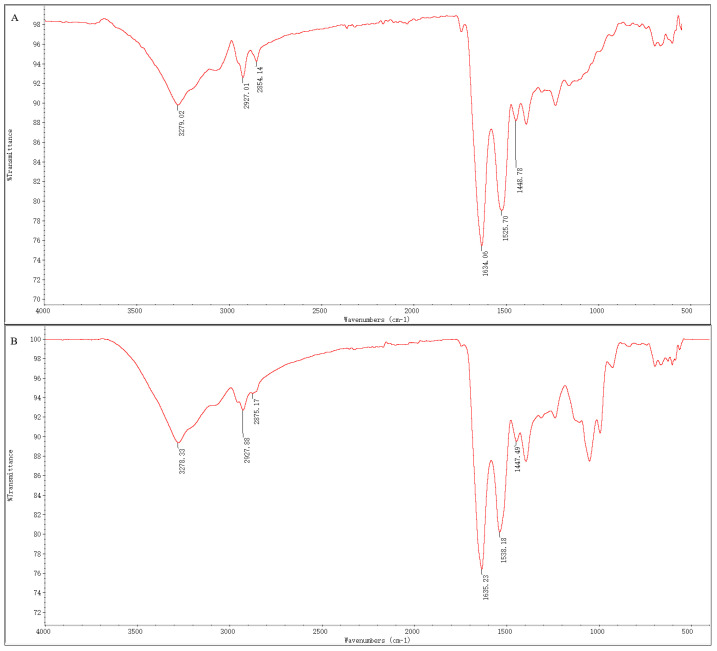
FTIR of protein powder: (**A**) is CPI; (**B**) is WS.

The amide groups of polypeptides and proteins possess nine characteristic vibrational modes or group frequencies, namely the amide I−VII band, amide A band, and amide B band [39,40]. The spectral region of the amide I band is between 1600–1700 cm^−1^, which is mainly the C=O stretch; the spectral region of the amide II band is between 1530–1550 cm^−1^, mainly the C-N stretch and N-H bend [40,41]. It can be seen from Figure 2 that the main characteristic peak ranges of the CPI and the WS are similar. There is a strong absorption peak near the region of 1635 cm^−1^ and 1530 cm^−1^, which indicates that both have the C=O, C-N stretch, and N-H bend. The C=C stretch at the range of 1450 cm^−1^ and the O-H stretch at the range of 3280 cm^−1^ show that there may also be an aromatic nucleus and hydrogen bonds in the two protein samples [42,43]. In addition, the FTIR analysis of the extracted protein freeze-dried samples show that two peaks, 2927 cm^−1^, 2854 cm^−1^, and 2928 cm^−1^, 2875 cm^−1^, exist at the fat characteristic peaks (near the range of 2924–2856 cm^−1^) of both the CPI and WS samples, indicating that the separated protein samples still contain a certain amount of fat [44].

The difference in the composition and content of a protein’s secondary structure will have an important impact on its stability. The secondary structure in *Corylus mandshurica Maxim* kernel proteins was analyzed with reference to the experiments of He et al. [45]. where the α-helix was located at 1650–1660 cm^−1^; the β-fold at 1610–1642 cm^−1^; the β-turn at 1660–1680 cm^−1^; the irregular curl at 1642–1650 cm^−1^; and the β-anti-parallel fold at 1680–1700 cm^−1^. The α-helix is the main ordered structure in the secondary structure, which is maintained by intramolecular hydrogen bonds, and the conformation is the most stable [43,46,47]; the β-sheet is the second most compact and conformationally stable. A total of two types are included, the β-parallel sheet and the β-anti-parallel sheet, of which the β-anti-parallel sheet structure is more stable [47]. The overall flexibility of the protein is better than that of the α-helix; therefore, it is more favorable for certain functional properties of the protein to be exerted [48]; The β-turn and random coil are disordered structures in the secondary structure, which are more sensitive to changes in external physical and chemical factors, and are more likely to cause changes in the protein [40,49].

As can be seen from Table 5, the sum of the two structures of the α-helix and β-fold is about 70%, which is the main secondary structure of the CPI and the WS, indicating that the structure of the extracted protein is more orderly. However, the amount of the α-helix of the CPI is slightly lower, the amount of the β-parallel sheet is slightly higher, and the amount of the β-anti-parallel sheet is more similar. The percentages of the β-turned and irregularly curled structures are similar in both proteins, and the sum of the contents is about 30%, indicating that both proteins could resist protein changes due to external factors to some extent.

#### 2.5.3. Intrinsic Fluorescence Analysis

**Figure 3:**
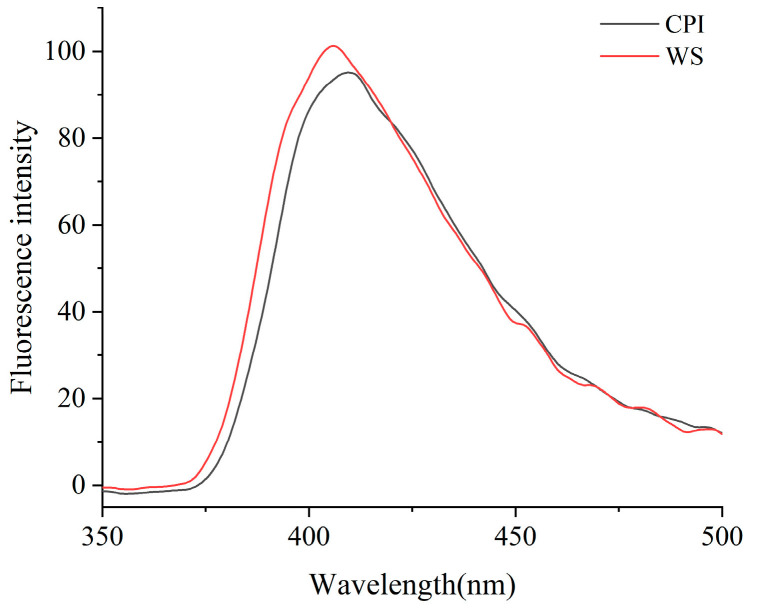
Fluorescence spectrum of *Corylus mandshurica Maxim* kernel proteins.

Due to the presence of aromatic amino acids such as phenylalanine, tryptophan, and tyrosine, the protein will produce fluorescence at a 280 nm or 295 nm excitation wavelength. Therefore, fluorescence spectroscopy could provide information to monitor conformational changes in protein tertiary structure [50]. The fluorescence intensity yields of the samples differ depending on the protein source. From Figure 3, it can be seen that there are some differences in the fluorescence spectra of the CPI and the WS. The maximum fluorescence intensity of the CPI is 95, corresponding to an emission maximum (λmax) value of 409 nm for the fluorescence spectrum; the maximum fluorescence intensity of the WS sample is 101, corresponding to a λmax value of 406 nm. In contrast, the CPI has a smaller fluorescence intensity, which may be due to the influence of acidic or alkaline solutions during the extraction process on the molecules of the CPI, and which led to extensive exposure of the tyrosine and tryptophan residues to the hydrophilic environment, which contributed to fluorescence quenching and resulted in a lower fluorescence intensity. The WS has lower λmax values, which indicates that the tryptophan residues in the protein are surrounded by a more hydrophobic environment [51]. Less exposure to tryptophan residues could also reduce protein aggregation via hydrophobic interactions [52].

#### 2.5.4. Microstructural Imaging Analysis

**Figure 4:**
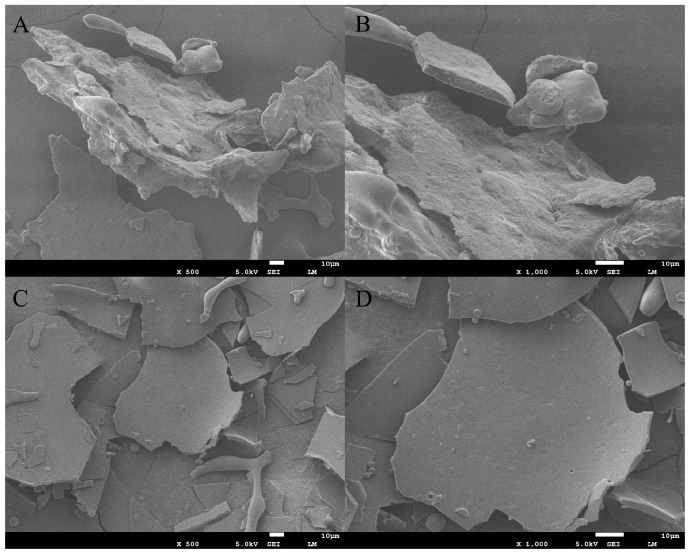
Scanning electron microscope image of protein powder: (**A**,**B**) is CPI; (**C**,**D**) is WS.

As can be seen in Figure 4, there are some differences in the microscopic morphology of the CPI and WS samples at a magnification of 500×. The WS samples are mainly regular flaky structures, accompanied by a part of some spherical structures with diameters of 1–10 μm and a small amount of rod-like structures with diameters of about 10 μm. The overall tissue structure is dense, with a smooth surface and few pores. The CPI samples are mainly an irregular flaky structure, which also contains some irregular rods and a small amount of spherical structures. The structure of the protein is dense, but the surface is rough and concave with pores. This may be because the alkaline extraction–isoelectric precipitation technique and isoelectric precipitation technique changed the microstructure of the *Corylus mandshurica Maxim* kernel proteins. The different microstructures also indicate the differences between the overall physicochemical and functional properties of different protein samples.

#### 2.5.5. Solubility Analysis

**Figure 5:**
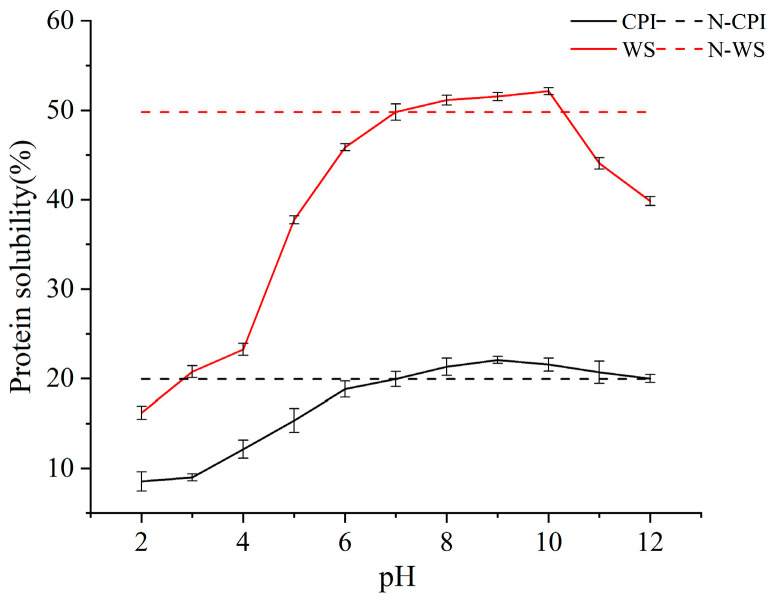
Solubility diagram of CPI and WS (Data results are expressed as triplicate means ± standard deviation).

After determination, it was found that the solubility of N-CPI was 19.96% and the solubility of N-WS was 49.79%. Both of them had good solubility and the WS showed obvious advantages. Since proteins are susceptible to certain changes in solubility due to different pH shifts during food processing, the solubility of the two proteins was investigated experimentally after different pH shifts. The results are shown in Figure 5. The solubility of both showed a trend of increasing and then decreasing under the offset treatment conditions with a pH range of 2–12. The solubility of the WS was higher than that of the CPI, and the difference in solubility between them gradually increased with increasing pH. The results showed that the maximum solubility of the CPI could be increased up to 22.08% (pH 9) and the maximum solubility of the WS could be increased up to 52.13% (pH 10). The increase in solubility was similar for both proteins, but the maximum solubility and the optimal pH were different. This indicates that the proteins have different structures and their properties are not the same.

## 3. Discussion

The protein content of the hazelnut kernels (21.89%) in this study was found to be higher than that of Chinese hazelnut (18.79%), Sichuan hazelnut (16.57%), hazelnut of Tieling Kaifuyuan (16.49%), and wild flat hazelnut of Changbai Mountain (17.4%), as well as that of Oregon hazelnuts (*Corylus avellana* L.) (14–18%) and Turkish hazelnut (*Corylus avellana* L.) (17.4–20.8%), and more similar to that of flat European hazelnut (22.74%), which is a high-protein hazelnut resource [53,54,55,56,57]. The quality of dietary protein is critical to its value, and the most important criterion for the potential of a protein source is its amino acid composition [58]. It was found that, unlike *Corylus* spp. in China studied by Jiang et al. [59], among the 17 common amino acids in the experimental hazelnuts, except for the AS which did not contain arginine, the other four proteins contained a complete set of 17 amino acids in abundance. Meanwhile, the content of each amino acid of the AS was low and differed greatly from that of the other four proteins, but the content of methionine + cystine was high. Methionine can participate in metabolism and synthesize cysteine in the body and can promote the synthesis of phosphatidylcholine, thus achieving the effect of preventing and treating fatty liver cirrhosis [60]. It is often used as a limiting amino acid in proteins such as soybeans, peanut milk, etc. [34]. To maximize the nutritional value of the food, it can be consumed together with *Corylus mandshurica Maxim* as a complementary or nutritional fortification. Glutamic acid, arginine, aspartic acid, and leucine in the remaining four proteins were the same as those in European hazelnut and Turkish hazelnut, and all of them accounted for a higher proportion of amino acids in hazelnut, and the proportion of glutamic acid and arginine in *Corylus mandshurica Maxim* was higher than one-third of the total amount of amino acids in each protein [25,57]. Although glutamate and arginine are non-essential amino acids, they play an important role in human growth and metabolism. Arginine is an essential amino acid to maintain the growth and development of infants and has been described as a “miracle molecule”, playing an important role in the synthesis of proteins, polyamines, and nitric oxide [61]. According to epidemiological investigation, eating nuts can reduce the incidence rate of coronary heart disease because L-arginine plays an anti-atherosclerosis role [62]. Glutamic is the main amino acid in cereal protein, which can activate the tricarboxylic acid cycle, and plays an important role in the nitrogen metabolism process of the body. As one of the most consumed amino acids in brain activity, it is of great significance for brain function and normal activities of the central nervous system. Further calculation of nutritional parameters revealed that four of the five proteins, except for the AS, had similar nutritive value and biomass price to the FAO/WHO model ratios, but only the WS and the CPI had more potential to replace plant proteins after considering the protein content issue.

The study of protein structure and properties is more helpful in promoting protein development and utilization. The molecular weight distribution pattern of the CPI and the WS in *Corylus mandshurica Maxim* is similar to that of European hazelnut, but the two hazelnut proteins differ in the distribution of substituent bands, and the overall distribution of small molecular weights suggests that hazelnut proteins may be easier to be digested and absorbed by the human body [54]. There are some differences in the surface structure of the two proteins: the surface of the WS is smoother and flatter and contains certain tiny spherical structures; combined with protein solubility, the protein with a flat and regular surface has a better solubility, which is consistent with the results of the study by Mao et al. [63]. Of the two proteins, WS has relatively high solubility. However, the small change in the solubility of the two proteins after pH adjustment should be closely related to the relatively high percentage of the α-helix and β-fold structures in the two proteins. It indicates that both proteins are more stable and easily resistant to conformational and structural changes that may occur during the processing and handling of the proteins as food products, thus retaining their nutritional or functional properties to a greater extent.

In this experiment, the basic nutritional value and structural properties of *Corylus mandshurica Maxim* kernel proteins were investigated to assess the potential of hazelnut as a novel plant protein resource, in order to provide a basis for the innovative utilization of *Corylus mandshurica Maxim* in food. In addition, the methods of amino acid analysis involved in this study, intrinsic fluorescence, FTIR, and microstructure imaging, have some advantages in studying the nutritional and structural changes of proteins. It can provide some basis for subsequent studies on the changes in physicochemical properties during the processing of *Corylus mandshurica Maxim* kernel proteins.

## 4. Materials and Methods

### 4.1. Materials

The *Corylus mandshurica Maxims* were purchased from the Xiaoxing’an Mountains of northeastern China, and the raw materials of the *Corylus mandshurica Maxim* kernel were obtained after manual shelling. The raw materials were soaked in 0.2% NaOH solution and stirred for 2 min before being retrieved, then pulled out, peeled off the seed coat and rinsed. Then, it was dried in an oven at 40 °C for 12 h until constant weight and finally stored at −20 °C for spare parts [64].

### 4.2. Chemicals and Reagents

Bovine serum albumin (BSA) and Marker for SDS–PAGE (molecular weight range 14.4 to 97.4 kDa) were purchased from Solarbio (Solarbio Science & Technology Co., Ltd., Beijing, China). All other chemicals used were of analytical grade.

### 4.3. Preparation of DCF

The *Corylus mandshurica Maxim* kernels, peeled and dried, were crushed in a pulverizer, added to a petroleum ether solution in a 1:6 ratio (*v*/*w*), stirred for 1.5 h at room temperature with a magnetic stirrer, and filtered to remove the organic solvent. The defatted procedure was conducted two times. The defatted powder was dried in an oven at 40 °C for 12 h until reaching constant weight and then passed through a 60 mesh sieve to obtain the DCF, which was stored at −20 °C and set aside [65].

### 4.4. Proximate Chemical Analysis of Corylus mandshurica Maxim Kernel and DCF

The protein content, fat content, moisture content, and ash content of the shelled *Corylus mandshurica Maxim* raw material was determined according to National Standards of China, GB 5009.5-2016 (Kjeldahl method), GB 5009.6-2016 (Soxhlet extraction method), GB 5009.3-2016 (drying method), and GB 5009.4-2016 (burning method), respectively. In addition, the purity of other protein samples was determined by the Kjeldahl method [66,67,68,69]. The protein conversion coefficient F is 5.30.

### 4.5. Preparation of CPI from DCF

The CPI from the DCF was prepared using the traditional alkaline dissolving and acid precipitating method, as described by Du [70] and Ma et al. [71] with some modifications. The DCF was dispersed in distilled water (solid: solvent = 1:10, *w*/*v*), and the pH of the dispersion was adjusted to 8.0 with 0.1 M NaOH. The resultant dispersion was gently stirred for 1 h at room temperature and centrifuged at 4000 r/min for 15 min. The process was repeated to fully extract the CPI. The protein from combined supernatants of the first and second extraction was isoelectrically precipitated at pH 4.5 with 0.1 M HCl and centrifuged at 4000 r/min for 15 min. The precipitate was washed and dissolved with distilled water and the pH was adjusted to 7.0, then it was freeze-dried and stored at −20 °C.

### 4.6. Preparation of Different Fractions of Proteins from DCF

**Figure 6:**
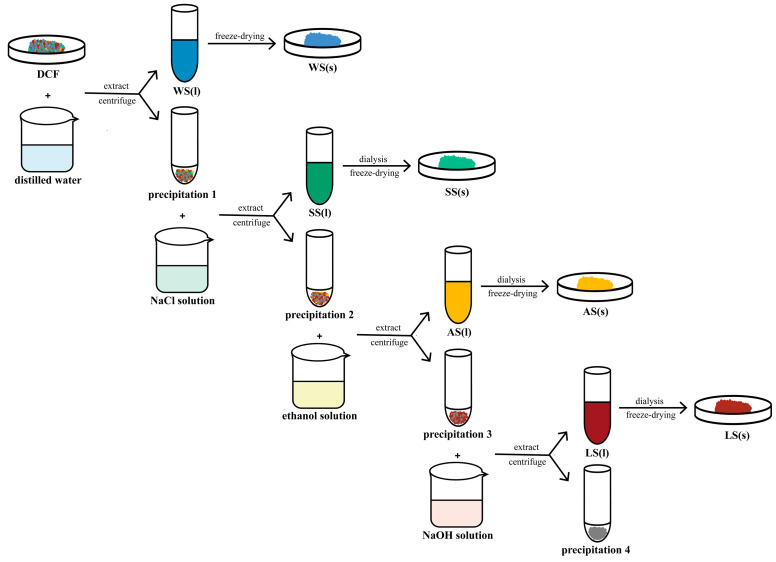
Preparation of different fractions of proteins from DCF.

*Corylus mandshurica Maxim* kernel proteins were fractionated from the DCF according to the Osborne differential extraction method as described by Siong [72], Du [70], and Zeng et al. [73] with some modifications. The flow of the experiment is shown in Figure 6. The DCF was dispersed in distilled water (solid: solvent = 1:10, *w*/*v*). The resultant dispersion was gently stirred for 1 h at room temperature and centrifuged at 4000 r/min for 15 min to produce the WS. The 0.5 M NaCl solution volume was added 10 times to the above precipitation, and the SS was extracted under the same conditions. The supernatant was concentrated and placed into a dialysis membrane for 36 h, then extracted and precipitated with 10 times the volume of 75% ethanol for 1 h, and centrifuged to obtain the AS. Finally, the LS was extracted with 10 times the volume of 0.1 M NaOH solution and dialyzed. To recover most of the proteins, each extraction step was performed twice and the respective extracts were pooled. The processed sample was placed into a closed plastic tube after freeze-drying and stored at −20 °C.

### 4.7. Determination of Corylus mandshurica Maxim Kernel Protein Contents and Amino Acid Compositions

The extracted CPI was combined with the four graded proteins to determine the protein content and calculate the extraction rate by the Coomassie Blue Method [74]. The amino acid composition was determined by GB 5009.124-2016 [75], using an L-8800 amino acid autoanalyzer to determine the amino acid composition in the five proteins. The results were reported as a percentage of amino acids mg per g of protein content. The ratio of EAA to TAA was reported as E/T(%).

(1) $$Extraction\;rate\left(\%\right)=\frac{Protein\;content\;in\;extracts\left(g\right)}{Total\;protein\;content\;in\;DCF\left(g\right)}\times 100\%$$

### 4.8. Evaluation of the Nutritional Value of Five Proteins in Corylus mandshurica Maxim Kernel

Based on the FAO/WHO reference pattern and the methods reported in the previous study, the nutritional quality of the amino acids was evaluated by AAS, CS, EAAI, BV, and NI [61,76,77,78]. Each of the indicators was calculated using the following formulas.

(2) $$AAS=\frac{Content\;of\;essential\;amino\;acids\;in\;the\;sample\;(mg/g\;protein)}{Content\;of\;essential\;amino\;acids\;in\;the\;standard\;model\;(mg/g\;protein)}\times 100\%$$

(3) $$CS=\frac{Content\;of\;essential\;amino\;acids\;in\;the\;sample\;(mg/g\;protein)}{Content\;of\;essential\;amino\;acids\;in\;the\;Egg\;standard\;model\;(mg/g\;protein)}\times 100\%$$

(4) $$EAAI=\sqrt[{n}]{\frac{{Lys}^{p}}{{Lys}^{s}}\times \frac{{Leu}^{p}}{{Leu}^{s}}\times \cdot \cdot \cdot \times \frac{{Trp}^{p}}{{Trp}^{s}}}\times 100$$

(5) BV=1.09×EAAI−11.70

(6) $$NI=\frac{EAAI\times the\;content\;of\;protein}{100}$$

where *n* was the number of essential amino acids; *p* was the amino acid content of the sample (mg/g); and *s* was the amino acid content of the egg protein (mg/g).

### 4.9. Structural Properties

#### 4.9.1. Determination of SDS–PAGE

Using pre-prepared 4% stacking gel and 15% separating gel solution, the protein was diluted to 2 mg/mL and added to the sample loading buffer at a ratio of 1:1, held for 5 min at 100 °C, centrifuged, and 10 μL was taken into the gel and electrophoresed. The electrophoresis was stopped when the bromophenol blue indicator ran to the bottom of the separation gel, and the electrophoresis gel was taken out and stained with Coomassie blue stain, decolorized, and photographed for analysis [79].

#### 4.9.2. Determination of FTIR

The freeze-dried WS and CPI samples were scanned and analyzed by FTIR in the range of 550–4000 cm^−1^. After the spectrum was automatically modified and corrected by EZ Omnic software (Version 7.3), the Gaussian deconvolution and the second derivation and fitting were performed on the amide I band (1600–1700 cm^−1^) in the FTIR using PeakFit v4.12 software [39]. Finally, the percentage of the sample’s secondary structure of the protein was calculated based on the peak area.

#### 4.9.3. Intrinsic Fluorescence

The samples were dissolved and centrifuged, and the supernatant was taken and diluted to a protein solution with a concentration of 0.2 mg/mL. Intrinsic fluorescence emission spectra were obtained by a fluorescence spectrophotometer (LS55, PE Inc., Waltham, MA, USA). After determining the optimal excitation wavelength of the solution for each protein, spectral acquisition was performed in the wavelength range of 300–560 nm. The slit of the excitation was 15 nm and the emission wavelength was set to 5 nm [80]. 

#### 4.9.4. Determination of Microstructural Imaging

The protein samples were imaged for microstructure after appropriate magnification using a scanning electron microscope (JSM-7500 F, Japan Electronics Co., Ltd. (JEOL), Tokyo, Japan) [81].

#### 4.9.5. Determination of Solubility

The solubility of the proteins was determined by the method of Wang et al. [82], with slight modifications. A certain amount of the sample was dispersed in distilled water at a ratio of 1:100 to prepare a solution of 10 mg/mL, the pH was adjusted to 7.0, and the samples were stirred with the magnetic force for 1 h at room temperature to fully hydrate, and then centrifuged at 8000 r/min for 10 min. The supernatant was suitably diluted and the soluble protein content was determined using the Coomassie Blue Method. To further study the relationship between the structure and solubility of proteins, the samples were treated with a pH 2.0–7.0 range offset to induce protein unfolding for a certain time, and then the pH was adjusted to 7 to restore its folding, and then its solubility was determined.

(7) $$Solubility\left(\%\right)=\frac{Soluble\;protein\;content(g)}{Total\;protein\;content(g)}\times 100$$

#### 4.9.6. Statistical Analysis

All experiments were performed in triplicate and the results are expressed as the mean ± standard deviation of three measurements.

## Figures and Tables

**Table 1:** The chemical composition of *Corylus mandshurica Maxim* kernel and DCF.

Name of Sample	Moisture (%)	Fat (%)	Protein (%)	Ash (%)
*Corylus mandshurica Maxim* kernel	5.51 ± 0.05	61.00 ± 0.69	21.89 ± 0.92	2.67 ± 0.11
DCF	3.49 ± 0.06	8.68 ± 0.28	49.81 ± 0.98	6.64 ± 0.13

**Table 2:** Amino acid composition and content of the five proteins in *Corylus mandshurica Maxim* kernel proteins.

Name of Amino Acid (mg/g pro)	Protein Fractions					FAO/WHO
	WS	SS	AS	LS	CPI	
Threonine (Thr)	25.67 ± 0.57	19.96 ± 0.97	1.61 ± 0.34	17.82 ± 0.77	24.27 ± 3.77	40
Valine (Val)	47.10 ± 0.35	38.23 ± 1.19	27.96 ± 0.94	39.24 ± 1.09	47.69 ± 1.21	50
Methionine + Methionine (Met + Cys)	56.78 ± 7.34	68.08 ± 7.46	144.83 ± 1.01	74.44 ± 4.55	39.89 ± 6.00	35
Isoleucine (Ile)	36.13 ± 0.47	26.44 ± 0.86	13.19 ± 1.20	24.88 ± 0.97	39.33 ± 0.96	40
Leucine (Leu)	64.68 ± 0.84	52.34 ± 0.99	17.32 ± 1.19	47.57 ± 0.85	71.35 ± 2.14	70
Phenylalanine + Tyrosine (Phe + Tyr)	71.12 ± 0.29	59.12 ± 1.91	32.40 ± 8.69	47.12 ± 2.70	76.09 ± 1.84	60
Lysine (Lys)	23.05 ± 0.06	30.57 ± 0.18	12.28 ± 0.72	21.00 ± 0.29	20.94 ± 0.74	55
Aspartic (Asp)	87.94 ± 1.94	54.44 ± 0.82	0.45 ± 0.73	33.25 ± 0.08	96.78 ± 3.46	
Serine (Ser)	38.72 ± 2.38	28.61 ± 0.24	1.64 ± 0.74	21.76 ± 0.68	45.24 ± 3.61	
Glutamic (Glu)	225.08 ± 2.52	170.75 ± 2.15	6.04 ± 2.65	62.20 ± 0.18	200.93 ± 7.30	
Glycine (Gly)	38.48 ± 0.41	32.43 ± 0.09	1.30 ± 1.08	19.74 ± 0.07	38.91 ± 1.58	
Alanine (Ala)	43.42 ± 0.94	32.66 ± 0.76	30.50 ± 3.67	30.36 ± 0.14	44.55 ± 1.71	
Histidine (His)	23.44 ± 0.07	23.52 ± 0.58	13.96 ± 2.93	17.94 ± 0.49	25.12 ± 0.93	
Arginine (Arg)	128.18 ± 0.73	106.26 ± 0.88	0.00	33.19 ± 0.14	132.21 ± 5.13	
Proline (Pro)	33.46 ± 0.72	41.16 ± 1.28	4.51 ± 4.04	32.82 ± 1.41	45.21 ± 3.34	
TAA	943.24	784.56	307.97	523.33	948.51	
EAA	324.52	294.73	249.58	272.08	319.56	
NEAA	618.72	489.83	58.39	251.26	628.95	
(EAA/TAA)/%	34.41	37.57	81.05	51.98	33.70	
(EAA/NEAA)/%	52.46	60.17	433.62	108.29	50.85	

WS is a water-soluble protein, SS is a salt-soluble protein, AS is an alcohol-soluble protein, LS is an alkali-soluble protein, and CPI is protein isolates of *Corylus mandshurica Maxim* kernel. Cysteine and tyrosine are converted from methionine and phenylalanine in the human body, so methionine and cysteine, phenylalanine, and tyrosine are often combined when calculating the content of essential amino acids. Data results are expressed as triplicate means ± standard deviation.

**Table 3:** AAS, CS, and essential amino acid index of *Corylus mandshurica Maxim* kernel proteins.

Protein Samples	Nutritional Parameters	Thr	Val	Met + Cys	Ile	Leu	Phe + Tyr	Lys
WS	AAS	64.16	94.20	162.22	90.31	92.40	118.53	41.91
	CS	54.61	71.36	99.61	66.90	75.21	76.47	32.93
SS	AAS	49.89	76.47	194.50	66.10	74.77	98.53	55.58
	CS	42.46	57.93	119.43	48.96	60.86	63.57	43.67
AS	AAS	4.02	55.92	413.79	32.96	24.74	54.01	22.33
	CS	3.42	42.36	254.08	24.42	20.14	34.84	17.54
LS	AAS	44.56	78.49	212.70	62.20	67.95	78.54	38.17
	CS	37.92	59.46	130.60	46.07	55.31	50.67	29.99
CPI	AAS	60.66	95.39	113.96	98.33	101.93	126.81	38.08
	CS	51.63	72.26	69.98	72.84	82.97	81.82	29.92

**Table 4:** EAAI, BV, and NI values of *Corylus mandshurica Maxim* kernel proteins.

Indicators	WS	SS	AS	LS	CPI
EAAI	72.19	65.27	30.21	58.59	69.74
BV	66.99	59.45	21.23	52.16	64.32
NI	55.78	7.92	0.49	2.50	41.68

**Table 5:** Secondary structure and content of CPI and WS.

Secondary Structure	α-Helix	Parallel β-Sheet	Anti-Parallel β-Sheet	β-Turn	Random Coil
CPI	22.73 ± 0.92	41.52 ± 0.76	7.59 ± 0.83	15.41 ± 0.64	12.75 ± 0.73
WS	24.58 ± 0.71	38.91 ± 0.64	7.60 ± 0.67	16.06 ± 0.92	12.84 ± 0.97

## Data Availability

All available data are contained within the article.

## Abbreviations

Defatted *Corylus mandshurica Maxim* kernel flours, DCF; protein isolates of *Corylus mandshurica Maxim* kernel, CPI; water-soluble protein, WS; salt-soluble protein, SS; alcohol-soluble protein, AS; alkali-soluble protein, LS; essential amino acid, EAA; the total amino acid, TAA; non-essential amino acid, NEAA; amino acid score, AAS; chemical score, CS; the essential amino acid index, EAAI; biological value, BV; nutrition index, NI; Fourier transform infrared spectroscopic, FTIR; natural protein isolates of *Corylus mandshurica Maxim* kernel, N-CPI; natural water-soluble protein, N-WS.
